# Supplementary material for: Engineered dityrosine-bonding of the RSV prefusion F protein imparts stability and potency advantages
Source: Nat Commun. 2024 Mar 14;15:2202. doi: 10.1038/s41467-024-46295-8 (PMC10940300; doi:10.1038/s41467-024-46295-8)
Supplement: Supplementary file 1 — Supplementary Information [file 41467_2024_46295_MOESM1_ESM.pdf]

Supplementary Material for:

Engineered dityrosine-bonding of the RSV prefusion F protein imparts stability and potency advantages.

Includes: Supplementary Figures and Supplementary Figure Legends for 1-4; Supplementary Methods for 1-4

Supplementary Figures and Figure Legends:

Supplementary Figure 1:

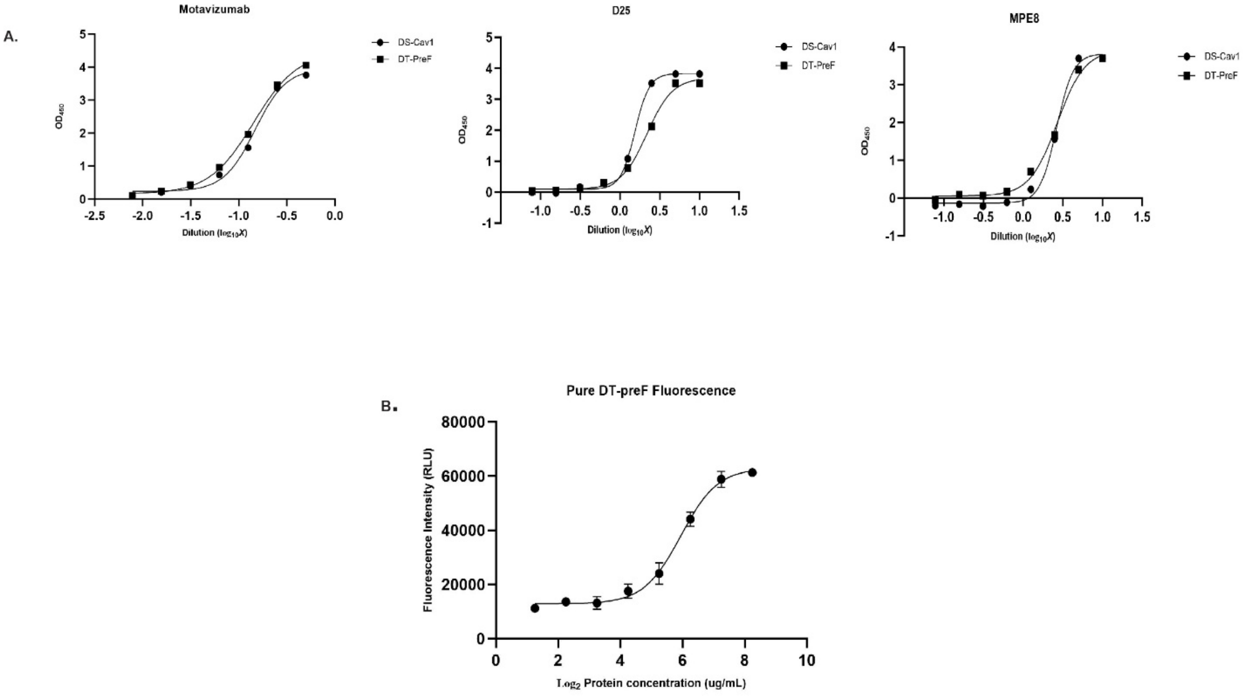

Supplementary Figure 1: Characterization of DT-preF by antigenic analysis and fluorimetry. (A) ELISA binding curves of DT-preF vs DS-Cav1 proteins using primary antibodies Motavizumab, D25, and MPE8 which are specific to epitopes in Site II, Site Ø, and Site III, respectively. (B) Protein concentration-based, fluorescence intensity analysis of crosslinked and purified DT-preF under native conditions. Data are presented as mean values +/- SD.

Supplementary Figure 2:

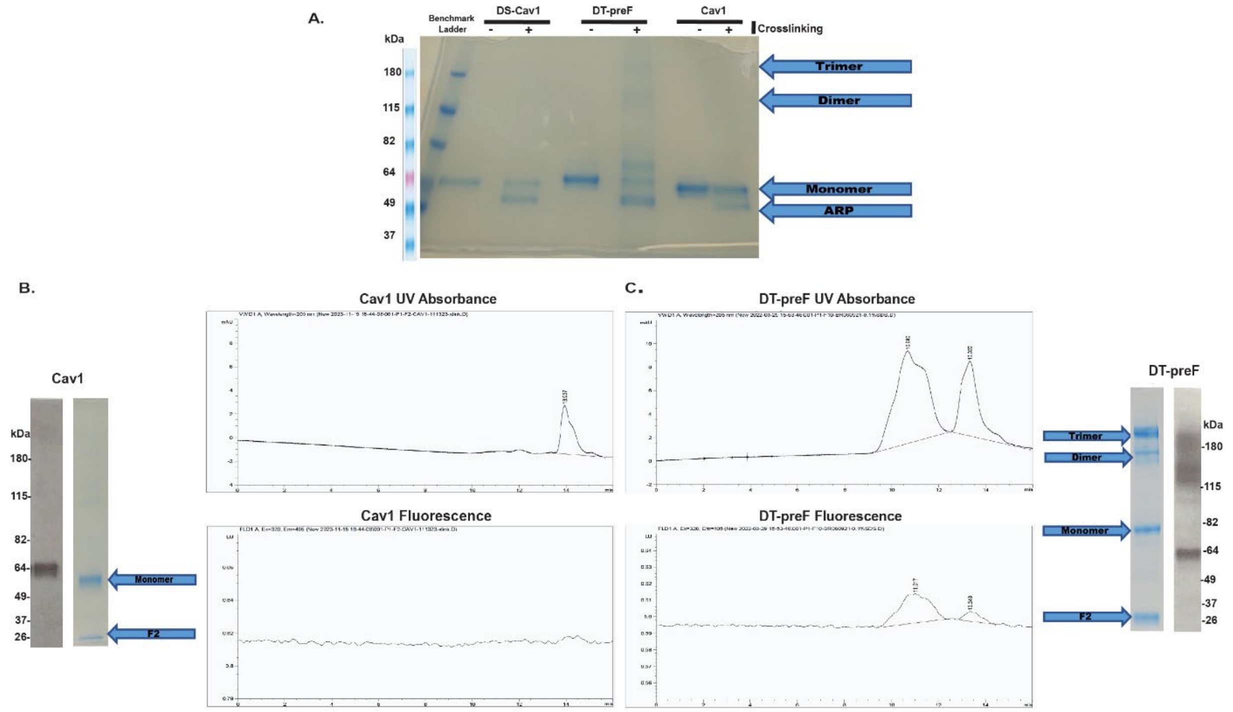

Supplementary Figure 2: Control protein analysis of DS-Cav1 and Cav1 exposed to crosslinking conditions. (A) Coomassie-stained SDS-PAGE gel analysis of crosslinking reactions pre and post crosslinking run under denaturing conditions for DS-Cav1, DT-preF and Cav1. Monomer, Dimer, trimer, and ARP protein bands are indicated. (B, left) Western Blot and Coomassie stained protein gel run under reducing conditions with purified Cav1 protein exposed to the crosslinking conditions. Monomer and F2 species are indicated by the arrow. (B, right) UV absorbance chromatogram (top) at 205 nm and fluorescence chromatogram (bottom) at Ex 320nm/ Em 405nm generated by SEC-HPLC under denaturing conditions. (C, right) Western Blot and Coomassie stained protein gel under reducing conditions for a representative DT-preF protein. Trimer, Dimer, Monomer and F2 species are indicated. (C, left) UV absorbance chromatogram (top) at 205 nm and fluorescence chromatogram (bottom) at Ex 320nm/ Em 405nm generated by SEC-HPLC under denaturing conditions.

Supplementary Figure 3:

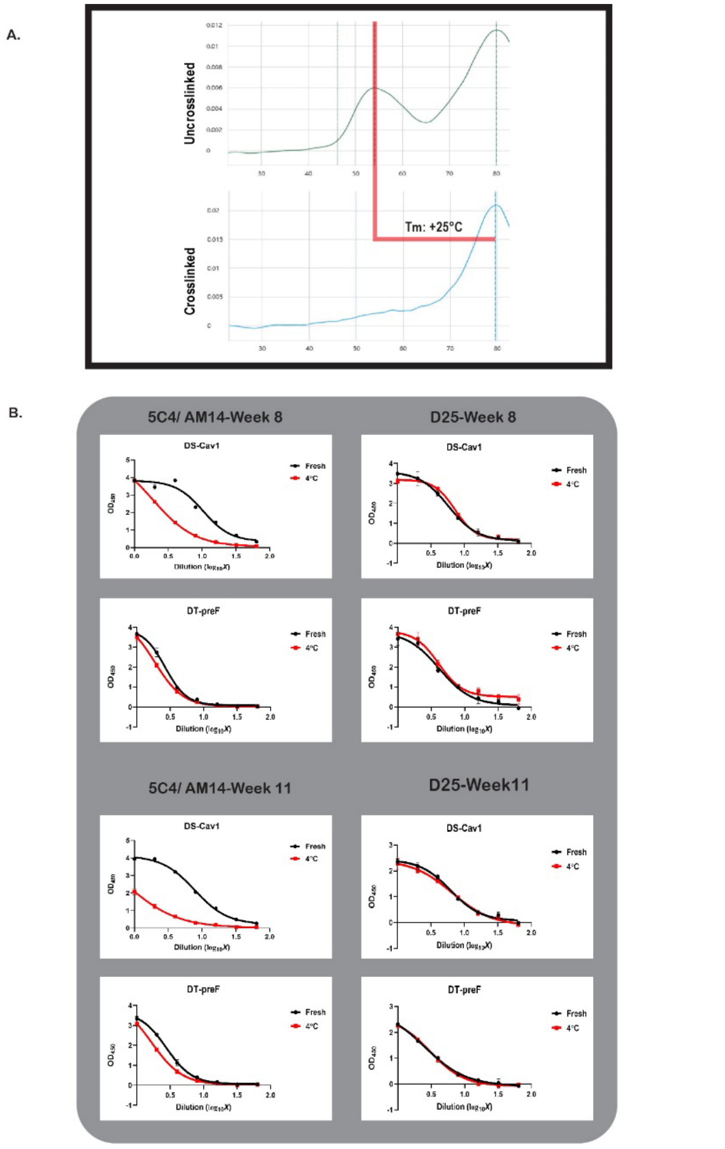

Supplementary Figure 3: Additional stability characterization of DT-preF. (A) Second derivative analysis of a Differential Scanning Fluorimetry (DSF) experiment of uncrosslinked ($preF^{C}$) and crosslinked (DT-preF). An increase of +25°C in the melting temperature is observed when DT-preF is analyzed and it ultimately reaches a melting temperature of 79.63°C as compared to uncrosslinked protein's melting temperature of 54.19°C. (B) ELISA-based stability analysis of dityrosine crosslinked DT-preF and DS-Cav1 proteins incubated at 4°C vs unincubated (fresh) proteins at the indicated timepoints. A highly prefusion-specific sandwich ELISA (5C4/ AM14) and D25 direct binding ELISA's were performed with binding curves presented on Motavizumab-normalized proteins.

Supplementary Figure 4:

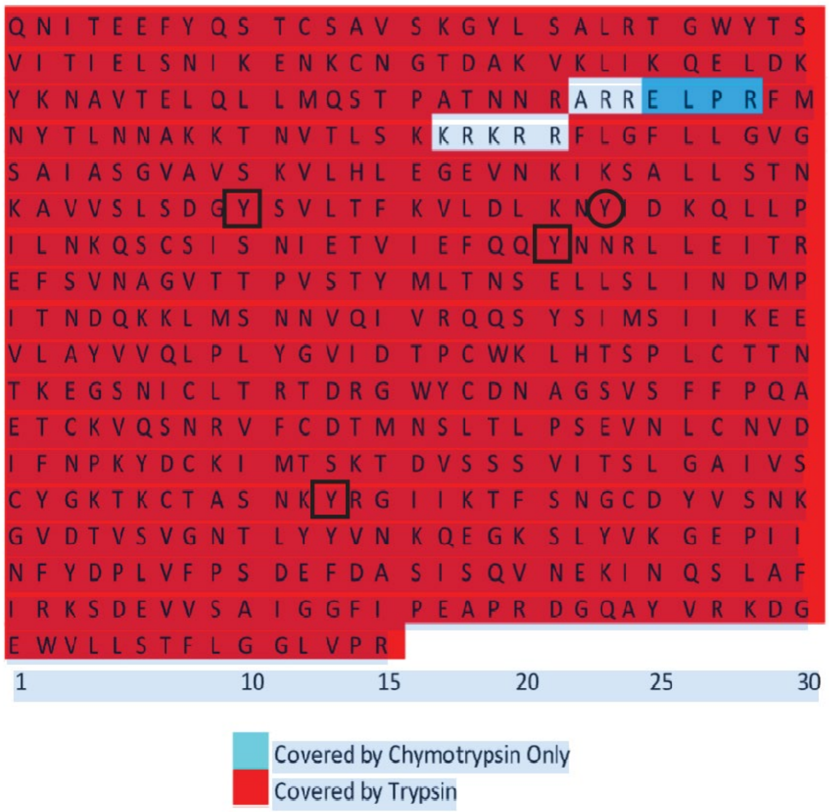

Supplementary Figure 4: Supp. Fig. 4: Coverage map of the uncrosslinked, mature $preF^{C}$ protein (Signal peptide and p27 region eliminated). Tyrosine residues involved in the dityrosine bond formation are indicated with a box (mutation) or a circle (endogenous tyrosine). The position of these residues is shifted from the GenBank sequence and correspond to Y160- Y403 and Y173 (endogenous)-Y201 in this amino acid sequence. The portion highlighted in red indicates the coverage area by Trypsin cleavage. and the region highlighted in blue indicates coverage by Chymotrypsin only cleavage.

Supplementary Methods:

Direct Binding ELISA Assay:

Purified DS-Cav1 and DT-preF antigens were coated on 96-Well Flat-Bottom Immuno PolySorp Plates (Thermo Fisher) at a starting concentration of 0.5ug/mL or 10ug/mL depending on the primary antibody used for probing. The plates were incubated at 37°C for 1h 40m or overnight at 4°C. Post- incubation and blocking, the plates were washed and incubated with Motavizumab at 5 ug/mL, D25 at 10ug/mL, or MPE8 at 10ug/mL, in 1% BSA, as the primary antibodies at 37°C for 1h. Post-incubation and extensive washing, the plates were incubated with anti-human

secondary antibody (Peroxidase AffiniPure $F(ab')_2$ Fragment Goat Anti-Human IgG, Jackson Immunochemicals) in 1% BSA at RT for 1h for the Motavizumab plate and 37°C for 1h for the D25 and MPE8 plates. The plates were finally washed extensively again, developed with 1-step TMB ELISA substrate solution (Thermo), stopped with Sulfuric Acid solution and read in a NOVOStar plate reader at 450nm wavelength.

Pure DT-preF Fluorescence Analysis:

Crosslinked protein purified as described in the materials and methods section was serially diluted in PBS in a black 96-well fluorescence plate. To maximize DT-specific fluorescence signal, 0.1 M NaOH was added to each well to a final concentration of 6.25 mM. Plates were read using fluorescence intensity measurement mode on a Novostar (BMG Labtech) plate reader with the PMT/ gain set to 4000. Plates were read in triplicate and nonlinear regression data analysis was performed using GraphPad PRISM version 10.1.1.

Nano Differential Scanning Fluorimetry (nanoDSF):

For DSF measurements, the Prometheus Panta instrument was used which measures changes in tryptophan fluorescence intensity and/or the ratio of tryptophan emission at 350 and 330 nm during unfolding of the protein. Tm was determined by taking the 2nd derivative of the F350/F330 ratios during the thermal shifts from folded to unfolded states of the protein. For analysis, samples were thawed at RT and run at a ramp rate of 1.5 ℃/min from 20 ℃ to 95 ℃ and fluorescence signals at 330 and 350 nm were collected for 500 ms per capillary in each round. Approximately 375 data points were collected.

Mass Spectrometry Analysis:

The LC Elution gradient parameters and instrument information for the mass spectrometry analysis are as follows:

HPLC: Agilent 1100 Binary pump

LC gradient: Total run time 200 min

Buffer A: 98%H2O 2% Acetonitrile with 0.025% TFA

Buffer B: 90% Acetonitrile, 10% H2O 0.025% TFA

Time (min)	%B	Flow rate (ml/min)
0	2%	0.06
20	2%	0.06
155	35%	0.06
165	90%	0.09
180	90%	0.11
182	2%	0.08
200	2%	0.06

Coverage map of the uncrosslinked mature preF^{C} is provided in Supplementary Figure 4.
