# Supplementary material for: Engineered dityrosine-bonding of the RSV prefusion F protein imparts stability and potency advantages
Source: Nat Commun. 2024 Mar 14;15:2202. doi: 10.1038/s41467-024-46295-8 (PMC10940300; doi:10.1038/s41467-024-46295-8)
Supplement: Supplementary file 3 — Reporting Summary [file 41467_2024_46295_MOESM3_ESM.pdf]

nature portfolio

Corresponding author(s): Mark Yondola, Ph.D.

Last updated by author(s): 02/08/2024

Reporting Summary

Nature Portfolio wishes to improve the reproducibility of the work that we publish. This form provides structure for consistency and transparency in reporting. For further information on Nature Portfolio policies, see our Editorial Policies and the Editorial Policy Checklist.

Statistics

For all statistical analyses, confirm that the following items are present in the figure legend, table legend, main text, or Methods section.

n/a	Confirmed	
☐	☒	The exact sample size (*n*) for each experimental group/condition, given as a discrete number and unit of measurement
☐	☒	A statement on whether measurements were taken from distinct samples or whether the same sample was measured repeatedly
☐	☒	The statistical test(s) used AND whether they are one- or two-sided<br>*Only common tests should be described solely by name; describe more complex techniques in the Methods section.*
☒	☐	A description of all covariates tested
☒	☐	A description of any assumptions or corrections, such as tests of normality and adjustment for multiple comparisons
☐	☒	A full description of the statistical parameters including central tendency (e.g. means) or other basic estimates (e.g. regression coefficient) AND variation (e.g. standard deviation) or associated estimates of uncertainty (e.g. confidence intervals)
☐	☒	For null hypothesis testing, the test statistic (e.g. *F*, *t*, *r*) with confidence intervals, effect sizes, degrees of freedom and *P* value noted<br>*Give P values as exact values whenever suitable.*
☒	☐	For Bayesian analysis, information on the choice of priors and Markov chain Monte Carlo settings
☒	☐	For hierarchical and complex designs, identification of the appropriate level for tests and full reporting of outcomes
☒	☐	Estimates of effect sizes (e.g. Cohen's *d*, Pearson's *r*), indicating how they were calculated

Our web collection on statistics for biologists contains articles on many of the points above.

Software and code

Policy information about availability of computer code

Data collection	No software was used.
Data analysis	GraphPad Prism Software Version 10.1.2 was used for statistical analysis. Mass Spectrometry data, for presentation, was prepared with Thermo XCalibur Qual Browser 3.1 Release. Prottech's in-house proprietary software was used to assign the mass spectrum.

For manuscripts utilizing custom algorithms or software that are central to the research but not yet described in published literature, software must be made available to editors and reviewers. We strongly encourage code deposition in a community repository (e.g. GitHub). See the Nature Portfolio guidelines for submitting code & software for further information.

Data

Policy information about availability of data

All manuscripts must include a data availability statement. This statement should provide the following information, where applicable:

- Accession codes, unique identifiers, or web links for publicly available datasets
- A description of any restrictions on data availability
- For clinical datasets or third party data, please ensure that the statement adheres to our policy

All data used to formulate this study are included in the attached source data file. Additionally, the mass spectrometry data have been deposited to the ProteomeXchange Consortium via the PRIDE partner repository with the dataset identifier PXD048897. The constructs designed in this study are based on the

GenBank sequence: 5W23_A and the crystal structures used for construct design are fully described in the cited references.

Human research participants

Policy information about studies involving human research participants and Sex and Gender in Research.

Reporting on sex and gender	N/A
Population characteristics	N/A
Recruitment	N/A
Ethics oversight	N/A

Note that full information on the approval of the study protocol must also be provided in the manuscript.

Field-specific reporting

Please select the one below that is the best fit for your research. If you are not sure, read the appropriate sections before making your selection.

☒ Life sciences ☐ Behavioural & social sciences ☐ Ecological, evolutionary & environmental sciences

For a reference copy of the document with all sections, see nature.com/documents/nr-reporting-summary-flat.pdf

Life sciences study design

All studies must disclose on these points even when the disclosure is negative.

Sample size

The number of mice used in Figures 5 and 6 are based on power calculations for the mixed model, where alpha = 0.05, power = 0.80 and correlations among repeated measures were 0.5. A sample size of 10 mice per group will provide enough power to detect a large between-group effect, along with a moderate effect within groups. Our preliminary studies show that the sample size is large enough to demonstrate significant outcome findings. We used 12 or 13 animals per group for the aged animal study to ensure a significant number made it to study completion.

For the cotton rat studies in Figure 7, it is assumed that there is a 10-fold difference between the groups in mean titer amounts to a difference of 1 in mean log (base 10)-transformed titer. With such a large expected difference, a relatively small sample size is required to achieve statistical significance. Specifically, if the standard deviation of the log (base 10)-transformed titer is 0.4, 5 cotton rats per group yields 80% power to detect a significant difference between groups at a two-sided 0.05 level of significance.

Data exclusions

In the murine immunization study of adult mice (Figure 6), the DT-preF group had one fewer animals than the other (9 vs 10) because one animal was found dead prior to the onset of vaccinations that was to be assigned to this group. In the aged animal study, there was not enough sample to run all assays from 4 animals in the aged, high dose, animal group. For these animals, ELISPOT assays were performed on 2 animals, while total serum analysis could only be performed for the other 2.

Replication

Neutralization titer (potency) analysis was performed in quadruplicate with assays run on different days and using at least 2 different frozen aliquots in the analysis. Aged animal neutralization titers were run in duplicate from a single serum aliquot due to serum constraints. Binding titer assays were performed in duplicate and the unvaccinated serum (prebleed) was used to subtract background binding from the terminal bleed values. All replication attempts were successful and are included in the analysis.

Randomization

- Animals were assigned randomly to groups to minimize bias
- Animals within a specific vaccination group were housed together to minimize chances of delivering the incorrect immunization
- Serum analysis was performed by interspersing serum from animals of different groups throughout all plates analyzed

Additional in vitro assays provided a direct comparison between 2 groups (DT-preF and DS-Cav1) and therefore randomization was not possible or required.

Blinding

To obtain the most accurate neutralization titer results, it is best to intersperse animals from different vaccination groups on each plate. In order to accomplish this, it was important to know the identity of the samples.

Reporting for specific materials, systems and methods

We require information from authors about some types of materials, experimental systems and methods used in many studies. Here, indicate whether each material, system or method listed is relevant to your study. If you are not sure if a list item applies to your research, read the appropriate section before selecting a response.

Materials & experimental systems

n/a	Involved in the study
☐	☒ Antibodies
☐	☒ Eukaryotic cell lines
☒	☐ Palaeontology and archaeology
☐	☒ Animals and other organisms
☒	☐ Clinical data
☒	☐ Dual use research of concern

Methods

n/a	Involved in the study
☒	☐ ChIP-seq
☒	☐ Flow cytometry
☒	☐ MRI-based neuroimaging

Antibodies

Antibodies used	Motavizumab-provided by Jason McLellan and purchased from Creative Biolabs (Cat#TAB-709, Lot#TAB-709-1805), AM14 provided by Jason McLellan and Barney Graham (VRC) and purchased from Creative Biolabs (Cat#PABL-321, Lot#CB0720Z06), D25 and MPE8 were provided by Jason McClellan and Barney Graham. 5C4, was provided by Barney Graham (VRC). Secondary Antibodies: • Peroxidase AffiniPure F(ab')$_2$ Fragment Goat Anti-Human IgG, Fcγ fragment specific, Jackson Immunochemicals; Catalog # 109-036-008, Amersham ECL HRP Conjugated Antibodies sheep anti-mouse, (Cytiva Life Sciences (Cat#NXA931-1mL), HRP Rat anti-mouse IgG2a, BD Pharmingen; Catalog #553391, Biotin-SP (long spacer) Affinipure goat anti-mouse IgG, Fc gamma fragment specific, Jackson Immunochemicals; Catalog #115-065-071,
Validation	Antibodies provided by Jason McLellan and Barney Graham (VRC) were not validated in-house but are described in the following publication-PLos Pathogens, 2015, Jul; 11(7): e1005035. Antibodies purchased from Creative Biolabs were validated by ELISA using pre and post-fusion antigens and compared side-by-side with VRC provided stocks. In addition, the antibodies were, "tested positive against native RSV" according to the manufacturer's landing page.

Eukaryotic cell lines

Policy information about cell lines and Sex and Gender in Research

Cell line source(s)	293Freestyle (ThermoFisher Cat#R79007), Hep-2 (ATCC, Cat#CCL-23-HeLa contamination reported by ATCC), 293T (in-house from Mount Sinai School of Medicine), Composite CHO expressing RSV F (Abzena, ltd)
Authentication	Direct purchase from ATCC for 293Freestyle, Hep-2 confirmed by morphology. Morphology only for 293T and CHO.
Mycoplasma contamination	293T cells were not tested for mycoplasma contamination, but Hep-2 and 293Freestyle were directly purchased from suppliers. The specified CHO cell line was confirmed to be Myco plasma free using the Invivogen Mycostrip mycoplasma detection kit (Cat#rep-mys-10).
Commonly misidentified lines (See ICLAC register)	Hep-2 ATCC provides a letter stating that there is HeLa contamination in their Hep-2 cell line upon purchase.

Animals and other research organisms

Policy information about studies involving animals; ARRIVE guidelines recommended for reporting animal research, and Sex and Gender in Research

Laboratory animals	Mice (Balb/c, CB6F1/J), cotton rats
Wild animals	No wild animal were used in the study.
Reporting on sex	For the in vivo stability and potency experiments in Figures 5B and 6A, female mice were used to inform our maternal to infant vaccination program. In the aged mouse study and the cotton rat study, both male and female animals were used.
Field-collected samples	No field collected samples were used in the study.
Ethics oversight	Experiments were performed utilizing NIH and United States Department of Agriculture guidelines, The Public Health Service Policy on Humane Care and Use of Laboratory Animals, and experimental protocols approved by the Baylor College of Medicine's Investigational Animal Care and Use Committee (IACUC; Protocol # AN-2307).

Note that full information on the approval of the study protocol must also be provided in the manuscript.
